# Survival outcomes of surgical and non-surgical treatment in elderly patients with stage I pancreatic cancer: A population-based analysis

**DOI:** 10.3389/fmed.2022.958257

**Published:** 2022-09-29

**Authors:** Duorui Nie, Qingxia Lan, Bin Shi, Fei Xu

**Affiliations:** ^1^College of Pharmacy, Hunan University of Chinese Medicine, Changsha, China; ^2^Graduate School, Hunan University of Chinese Medicine, Changsha, China; ^3^First Clinical Medical College, Guangzhou University of Chinese Medicine, Guangzhou, China; ^4^Hunan Engineering Technology Research Center for Bioactive Substance Discovery of Chinese Medicine, Changsha, China; ^5^Hunan Province Sino-US International Joint Research Center for Therapeutic Drugs of Senile Degenerative Diseases, Changsha, China

**Keywords:** elderly, pancreatic cancer, SEER, surgery, chemotherapy, radiation

## Abstract

**Background:**

Due to underrepresentation in randomized controlled trials among old people (≥65 years old), the effectiveness of clinical trial-based recommendations about the treatment for stage I pancreatic cancer remains controversial. In this research, we intended to investigate the different strategies of this population in surgery group and non-surgery group.

**Materials and methods:**

Elderly patients aged 65 years or older with histologically diagnosed stage I pancreatic cancer from 2006 to 2017 were identified from the Surveillance, Epidemiology, and End Results (SEER) database. The included patients were divided into surgery group (receiving surgery with chemotherapy or chemoradiotherapy) and non-surgery group (receiving radiotherapy, chemotherapy, both, or neither). Overall survival (OS) and cancer-specific survival (CSS) were compared between groups by Kaplan–Meier analysis. Cox proportional hazards regression (Cox) proportional hazards regression was used to determine factors associated with survival.

**Results:**

A total of 2,448 eligible patients were recruited. Among them, 18.4% were treated surgically and 81.6% were treated non-surgically. The median OS (mOS) was 26 months (95% CI: 24–30 months) in the surgery group and 7 months (95% CI: 7–8 months) in the non-surgery group. In multivariate analyses, surgery was an important factor in improving OS compared with non-surgical treatment (HR: 0.34, 95% CI: 0.29–0.39, *p* < 0.001). In subgroup analysis, surgery plus chemotherapy was an independent factor for OS in the surgery group, while chemoradiotherapy, chemotherapy, and radiotherapy were independent prognostic factors for patients in the non-surgery group.

**Conclusion:**

Surgical resection and post-operative chemotherapy are recommended for elderly patients with stage I pancreatic cancer who can tolerate treatment, but post-operative chemoradiotherapy does not bring survival benefits compared with post-operative chemotherapy. Moreover, radiotherapy, chemotherapy, or the combination of radiotherapy and chemotherapy are significantly related to the prognosis of elderly patients with untreated pancreatic cancer, but chemoradiotherapy has the most obvious benefit.

## Introduction

As an extremely deadly malignant tumors, pancreatic cancer (PC) causes a large number of morbidity and mortality worldwide. Approximately 62,210 new pancreatic cancers and 49,830 pancreatic cancer-related deaths have been expected in 2022 (data come from the American Cancer Society) (1). Besides, the 5-year survival rate is low, which is only 20% even for resectable/locally pancreatic cancer in the early stage (2). Moreover, the progress of immunotherapy and targeting pancreatic cancer has not yet made a breakthrough and it is still being explored in difficulties. Surgery is still considered as the only potential treatment to achieve a cure for the patients in early stage, and to delay recurrence and improve survival, post-operative adjuvant chemotherapy seems necessary (3–5).

Seniors are the fastest-growing population in the United States, and by 2040, those 65 and older are projected to make up more than 20% of the total population (6). Pancreatic cancer occurs primarily in the elderly population, with approximately 67% of pancreatic cancers reported to occur in patients 65 years of age and older, and this number is increasing (7, 8). For elderly individuals with surgically resectable pancreatic cancer (such as stage I), the initial management choice is whether to consider surgery or not. Although there is a lot of evidence that surgery, at an advanced age, is not absolutely contraindicated (9, 10). But older patients are generally less likely to be treated (11) and have a higher prevalence of comorbidity and a higher risk of treatment-related complications (12) than younger patients, which may be related to a lower chance of undergoing pancreatectomy (13), making decisions about surgery and post-operative adjuvant therapy difficult and complex. Furthermore, the elderly has been usually excluded in many previous studies, and it is not clear whether the same treatment offered to young patients is appropriate for elderly patients (14). Therefore, we queried the SEER database to obtain sufficient cases, aiming to determine: (1) whether the elderly with stage I PC can benefit from the surgery and further adjuvant chemotherapy is needed; and (2) which type of treatment may best benefit the elderly who fail to receive surgery.

### Patient population

The SEER database contains incidence data and tumor clinicopathological information from 18 population-based cancer registries covering nearly 27.8% of the U.S. population (15). The elderly patients with stage I PC were retrieved from 17 registries of the SEER Research Plus Data (2000–2019), which was submitted in November 2021, by using SEER*Stat 8.39 software. Patients meeting the following criteria were included: (1) the patient was diagnosed with the International Classification of Diseases for Oncology, Third Edition (ICD-O-3), site code: C25.0–C25.3, C25.5–C25.9 and histology code: 8140/3: Adenocarcinoma, NOS, 8480/3: Mucinous adenocarcinoma, 8481/3: Mucin-producing adenocarcinoma, 8500/3: Infiltrating duct carcinoma, NOS; (2) diagnosis was made between 2006 and 2017; (3) had 7th American Joint Committee on Cancer (AJCC) staging system stage I disease (T1-2N0M0); (4) age at diagnosis ≥ 65; (5) only one primary tumor; and the following patients were excluded: (1) unknown clinical information, such as T stage, race, surgery, and survival months; (2) receiving pre-operative treatment; (3) survival months are less than one month, and (4) patient died prior to recommended surgery.

### Covariates and endpoints

The study contains a series of variables as follows: gender, age at diagnosis, race, primary site, grade, years of diagnosis, primary site, 7th AJCC stage, radiotherapy, chemotherapy, primary site surgery, sequence of surgery and radiotherapy, sequence of surgery and chemotherapy, survival months, and terminal event state. The primary endpoint was OS, defined as death from any cause from the date of cancer diagnosis. The secondary endpoint is cancer-specific survival (CSS)—the time ranging from the date of diagnosis until the date of death due to pancreatic cancer.

### Statistical analysis

Categorical variables were described by frequencies and percentages, while continuous variables were described by medians and interquartile ranges. Categorical variables were measured by chi-square tests and continuous variables were compared by the Wilcoxon–Mann–Whitney test. The cox proportional hazards regression (Cox) proportional risk model was used to evaluate variables that had independent predictors of CSS and OS. Only variables significantly in the univariate Cox analysis were included in the multivariate Cox analysis. Hazard ratios (HRs) and 95% confidence intervals (CIs) were also estimated using Cox proportional hazards models. When subgroup analyses were taken into account, the multivariate models adjusted for demographic variables (Model I: age, sex, race, year of diagnosis); tumor-related variables (Model II: tumor site, grade, T stage); and all of these variables plus surgery methods/reason of no surgery (Model III).

A 1:1 propensity score matching method (PSM) with a caliper size of 0.2 was performed to balance the differences in baseline data between groups (16). Score between groups was calculated through logistic regression modeling based on the following 8 covariates: age, sex, race, grade, location, T stage, chemotherapy, and radiation. The survival curves were calculated by Kaplan–Meier method, and the difference in survival curves was tested by the Log-rank test. All statistical tests were performed using R statistical software (Version 4.0.4; R Foundation for Statistical Computing, Vienna, Austria^1^), and the following software packages are as follows: “tableone,” “survival,” “survminer,” and “MatchIt.” The statistical test was two sided and *p* < 0.05 was considered statistically significant.

### Patient characteristic

A total of 2,448 eligible patients were recruited. The clinical characteristics of the patients are shown in Table 1. There were 1,395 women and 1,053 men with a median age of 78 years. The proportions of white, black, and other races were 81.1%, 9.9%, and 9.0%, respectively. The most common site of primary tumor was the head of the pancreas (65.6%), followed by the body and tail of the pancreas (16.7%). Regarding the distribution of treatment, 451 (18.4%) patients underwent surgical treatment and 1,997 (81.6%) underwent non-surgical treatment. The percentage of surgical was 55.0, 39.7, and 5.3% in patient aged 65–74, 75–84, and ≥85 years old, respectively (*p* < 0.001). The most common surgical procedure was pancreaticoduodenectomy (290/64.3%), followed by distal pancreatectomy (94/20.8%). The reasons why 1,997 patients with stage I did not undergo surgery were analyzed. A total of 1,498 patients (61.2%) did not recommend surgery, 236 patients (9.6%) had surgery contraindications, 87 patients (3.6%) refused surgery for unknown reasons, and 176 patients (7.2%) refused surgery. The surgery group was more likely to receive chemotherapy (50.6 vs. 41.8%) than radiation (14.4 vs. 21.7%). Additionally, the two groups showed significant differences in other clinical characteristics. The clinical characteristics after adjustment for confounding by PSM are detailed in Supplementary Table 1.

**TABLE 1:** Clinical characteristics of surgical and non-surgical groups in elderly patients with stage I pancreatic cancer.

Variables	Overall, *N* (%)	No surgery, *N* (%)	Surgery, *N* (%)	*P*-value
All patients	2,448 (100%)	1,997 (81.6)	451 (18.4)	
Age (median [IQR])	78.0 [72.0, 84.0]	79.0 [72.0, 85.0]	74.0 [69.0, 78.0]	<0.001
**Age at diagnosis**				
65–74	889 (36.3)	641 (32.1)	248 (55.0)	<0.001
75–84	1,018 (41.6)	839 (42.0)	179 (39.7)	
85+	541 (22.1)	517 (25.9)	24 (5.3)	
**Sex**				
Female	1,395 (57.0)	1,158 (58.0)	237 (52.5)	0.04
Male	1,053 (43.0)	839 (42.0)	214 (47.5)	
**Race**				
Black	242 (9.9)	210 (10.5)	32 (7.1)	0.039
Other	221 (9.0)	172 (8.6)	49 (10.9)	
White	1,985 (81.1)	1,615 (80.9)	370 (82.0)	
**Year of diagnosis**				
2006–2011	903 (36.9)	706 (35.4)	197 (43.7)	0.001
2012–2017	1,545 (63.1)	1,291 (64.6)	254 (56.3)	
**Primary site**				
Body/Tail	410 (16.7)	285 (14.3)	125 (27.7)	
Head	1,703 (69.6)	1,439 (72.1)	264 (58.5)	
Other	335 (13.7)	273 (13.7)	62 (13.7)	
**Grade**				
I/II	553 (22.6)	266 (13.3)	287 (63.6)	<0.001
III/IV	288 (11.8)	182 (9.1)	106 (23.5)	
Unknown	1,607 (65.6)	1,549 (77.6)	58 (12.9)	
**T stage**				
T1	519 (21.2)	324 (16.2)	195 (43.2)	<0.001
T2	1,929 (78.8)	1,673 (83.8)	256 (56.8)	
**Chemotherapy**				
Yes	1,063 (43.4)	835 (41.8)	228 (50.6)	0.001
None	1,385 (56.6)	1,162 (58.2)	223 (49.4)	
**Radiation**				
Yes	499 (20.4)	434 (21.7)	65 (14.4)	0.001
None	1,949 (79.6)	1,563 (78.3)	386 (85.6)	
**Therapy**				
CRT	310 (12.7)	310 (15.5)		<0.001
CT	525 (21.4)	525 (26.3)		
RT	124 (5.1)	124 (6.2)		
None	1,038 (42.4)	1,038 (52.0)		
Surgery	221 (9.0)		221 (49.0)	
S + CRT	65 (2.7)		65 (14.4)	
S + CT	165 (6.7)		165 (36.6)	
*Reasons of no surgery				
Not recommended	1,498 (75.0)	1,498 (75.0)		
Contraindicated due to other cond	236 (11.8)	236 (11.8)		
Patient refused	176 (8.2)	176 (8.8)		
Unknown reason	87 (4.4)	87 (4.4)		
*Operation method				
Distal pancreatectomy	94 (3.8)		94 (3.8)	
Partial pancreatectomy	290 (11.8)		290 (11.8)	
Total pancreatectomy	53 (2.2)		53 (2.2)	
Unknown	14 (0.6)		14 (0.6)	

*The proportion of each part in no surgery and surgery are in parentheses.

### Survival analysis

**FIGURE 1:**
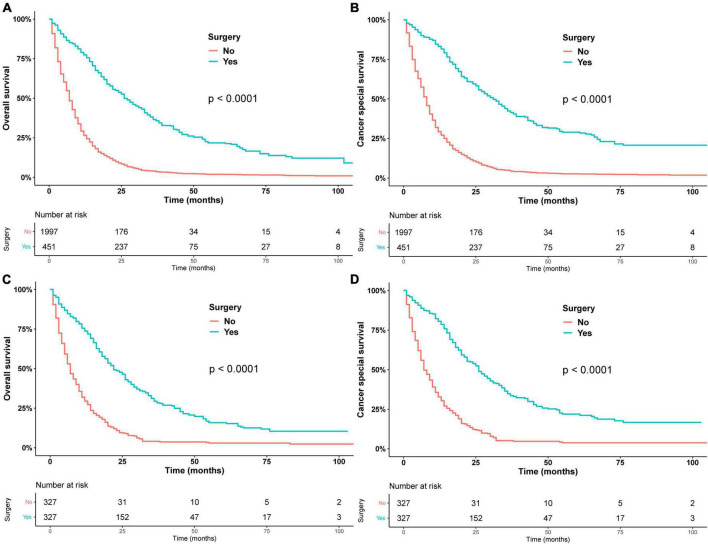
Overall survival in patients who underwent surgery or did not undergo surgery before PSM **(A)** and after PSM **(C)**. Cancer-specific survival in patients who underwent surgery or did not undergo surgery before PSM **(B)** after PSM **(D)**.

In general, patients who underwent surgery had better OS and CSS than the non-surgery group (Figure 1). The median OS (mOS) was 26 months (95% CI: 24–30 months) in the surgery group and 7 months (95% CI: 7–8 months) in the non-surgery group. After propensity matching analysis, the median OS was 22 months (95% CI: 20–26) in the surgery group and 7 months (95% CI: 7–8) in the non-surgery group. Table 2 shows the results of univariate and multivariate Cox regression. Surgery (HR = 0.34, 95% CI: 0.29–0.39), radiotherapy (HR = 0.81, 95% CI: 0.73–0.90), and chemotherapy (HR = 0.64, 95% CI: 0.58–0.70) were identified as a favorable prognostic factor, while older age (HR = 1.12, 95% CI: 1.02–1.23, HR = 1.13, 95% CI: 1.00–1.27), pancreatic head tumor (HR = 1.26, 95% CI: 1.12–1.41), and stage III/IV disease (HR = 1.30, 95% CI: 1.12–1.52) were harmful factors for OS, and similar results were found in CSS, except that 2012–2017 (HR = 0.86, 95% CI: 0.79–0.95) was identified as a favorable factor for CSS. The univariate and multivariate of OS and CSS after PSM are shown in Supplementary Table 2. Similarly, surgery was also identified as independent prognostic factor.

**TABLE 2:** Univariate and multivariate COX analyses for elderly patients with stage I pancreatic cancer.

Variables	OS				CSS			
	Univariate		Multivariate		Univariate		Multivariate	
	HR (95% CI)	*P*-value	HR (95% CI)	*P*-value	HR (95% CI)	*P*-value	HR (95% CI)	*P*-value
**Age at diagnosis**								
65–74	Reference		Reference		Reference		Reference	
75–84	1.30 (1.19–1.43)	<0.001	1.12 (1.02–1.23)	0.022	1.34 (1.21–1.47)	0.001	1.15 (1.04–1.27)	0.008
85+	1.69 (1.51–1.89)	<0.001	1.13 (1.00–1.27)	0.046	1.75 (1.56–1.96)	<0.001	1.16 (1.03–1.31)	0.018
**Sex**								
Female	Reference				Reference			
Male	0.97 (0.89–1.05)	0.476	/	/	0.96 (0.88–1.05)	0.416	/	/
**Race**								
Black	Reference		Reference		Reference		Reference	
Other	0.81 (0.67–0.98)	0.027	0.85 (0.70–1.03)	0.090	0.81 (0.66–0.98)	0.034	0.85 (0.69–1.04)	0.108
White	0.88 (0.77–1.01)	0.076	0.94 (0.82–1.08)	0.416	0.91 (0.78–1.05)	0.182	0.97 (0.84–1.12)	0.689
**Year of diagnosis**								
2006–2011	Reference				Reference		Reference	
2012–2017	0.92 (0.85–1.00)	0.065	/	/	0.91 (0.83–1)	0.038	0.86 (0.79–0.95)	0.002
**Primary site**								
Body/Tail	Reference		Reference		Reference		Reference	
Head	1.39 (1.24–1.56)	<0.001	1.26 (1.12–1.41)	0.000	1.43 (1.27–1.62)	<0.001	1.27 (1.12–1.43)	<0.001
Other	1.25 (1.08–1.46)	0.004	1.23 (1.06–1.43)	0.008	1.26 (1.07–1.48)	0.005	1.22 (1.03–1.43)	0.018
**Grade**								
I/II	Reference		Reference		Reference		Reference	
III/IV	1.51 (1.30–1.76)	<0.001	1.30 (1.12–1.52)	0.001	1.59 (1.36–1.86)	<0.001	1.36 (1.16–1.60)	<0.001
Unknown	1.98 (1.79–2.20)	<0.001	1.05 (0.93–1.18)	0.452	2.08 (1.86–2.32)	<0.001	1.06 (0.93–1.2)	0.382
**T stage**								
T1	Reference		Reference		Reference		Reference	
T2	1.85 (1.66–2.05)	<0.001	1.56 (1.40–1.75)	<0.001	1.94 (1.74–2.18)	<0.001	1.61 (1.43–1.81)	<0.001
**Surgery**								
None	Reference		Reference		Reference		Reference	
Yes	0.30 (0.27–0.34)	<0.001	0.34 (0.29–0.39)	<0.001	0.27 (0.23–0.3)	<0.001	0.30 (0.25–0.35)	<0.001
**Chemotherapy**								
None	Reference		Reference		Reference		Reference	
Yes	0.62 (0.57–0.68)	<0.001	0.64 (0.58–0.70)	<0.001	0.63 (0.58–0.69)	<0.001	0.66 (0.60–0.72)	<0.001
**Radiation**								
None	Reference		Reference		Reference		Reference	
Yes	0.84 (0.76–0.93)	0.001	0.81 (0.73–0.90)	<0.001	0.84 (0.75–0.93)	0.001	0.79 (0.70–0.88)	<0.001

**FIGURE 2:**
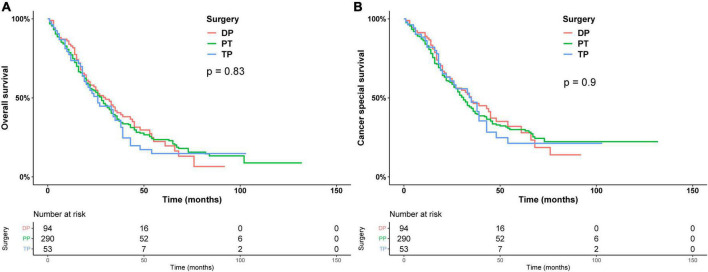
Kaplan–Meier analysis of overall survival **(A)** and cancer-specific survival **(B)** of different types of surgery.

**FIGURE 3:**
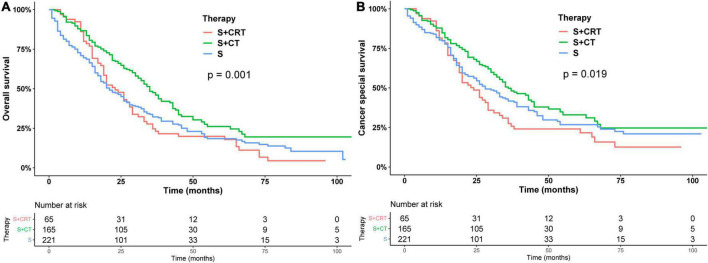
Kaplan–Meier analysis of overall survival **(A)** and cancer-specific survival **(B)** of post-operative adjuvant therapy.

Subsequently, we stratified by surgical and non-surgical treatment. In the subgroup of patients who underwent surgery, the median OS of patients who underwent partial pancreatectomy/pancreaticoduodenectomy (PT), distal pancreatectomy (DP), and total pancreatectomy (TP) were 28, 30, and 26 months (*p* = 0.83), (Figure 2A) and the median CSS was 35, 31, and 35 months (*p* = 0.9) (Figure 2B). Regarding post-operative adjuvant therapy, the post-operative chemoradiotherapy group and surgery-only group had similar prognosis, with median OS of 23 months, 21 months, and 5-year OS of 19.9 and 18.5%, respectively (*p* = 0.728), while the post-operative chemotherapy group had a better prognosis, with median OS of 35 months and 5-year OS of 26.1% (*p* < 0.01, Figure 3A). When adjusting for confounding factors, surgery plus chemotherapy was a favorable prognostic factor for OS in models I (HR = 0.69, 95% CI: 0.54–0.88), II (HR = 0.62, 95% CI: 0.48–0.79), and III (HR = 0.65, 95% CI: 0.51–0.84) (Table 3). The same results were obtained when the CSS was computed (Figure 3B). Surgery plus chemotherapy significantly improves outcomes in model II (HR = 0.72, 95% CI: 0.55–0.94) (Table 3).

**TABLE 3:** Cox regression analysis of the relationship of treatment modality with overall survival (OS) and cancer-specific survival (CSS).

End point	Subgroup	Model I		Model II		Model III	
		HR (95% CI)	*P*-value	HR (95% CI)	*P*-value	HR (95% CI)	*P*-value
OS	**Surgery group**						
	Surgery	1					
	Surgery + CT	0.69 (0.54–0.88)	0.003	0.62 (0.48–0.79)	<0.001	0.65 (0.51–0.84)	0.001
	Surgery + CRT	1.01 (0.74–1.37)	0.975	0.84 (0.62–1.15)	0.278	0.81 (0.59–1.13)	0.216
	**Non-surgery**						
	None						
	RT	0.66 (0.54–0.80)	<0.001	0.65 (0.54–0.78)	<0.001	0.64 (0.53–0.77)	<0.001
	CT	0.62 (0.55–0.69)	<0.001	0.57 (0.52–0.64)	<0.001	0.58 (0.52–0.65)	<0.001
	CRT	0.51 (0.44–0.58)	<0.001	0.49 (0.43–0.56)	<0.001	0.49 (0.43–0.56)	<0.001
CSS	**Surgery group**						
	Surgery						
	Surgery + CT	0.80 (0.61–1.05)	0.109	0.72 (0.55–0.94)	0.017	0.76 (0.57–1.00)	0.052
	Surgery + CRT	1.16 (0.83–1.63)	0.375	0.96 (0.69–1.35)	0.831	0.92 (0.65–1.31)	0.646
	**Non-surgery**						
	None						
	RT	0.63 (0.52–0.77)	<0.001	0.62 (0.51–0.76)	<0.001	0.61 (0.50–0.75)	<0.001
	CT	0.62 (0.55–0.69)	<0.001	0.57 (0.51–0.64)	<0.001	0.58 (0.52–0.65)	<0.001
	CRT	0.51 (0.44–0.58)	<0.001	0.49 (0.43–0.56)	<0.001	0.49 (0.43–0.56)	<0.001

**FIGURE 4:**
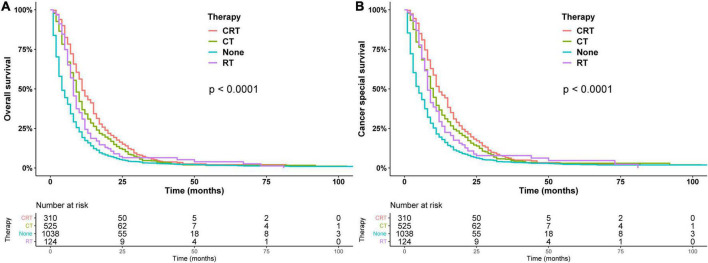
Overall survival **(A)** and cancer-specific survival **(B)** estimated by the Kaplan–Meier method in patients treated non-surgically.

In addition, the survival curves of patients who did not receive surgery are shown in Figure 4. In the patients who did not receive surgery, the median OS of chemoradiotherapy, chemotherapy, radiotherapy, and untreated patients were 11, 9, 8, and 5 months, respectively, and the 1-year OS were 48.1, 36.7, 29.3, and 19.5%, respectively (*p* < 0.01), chemotherapy and radiotherapy had similar prognosis (*p* = 0.22). RT, CT, and CRT had a better prognosis than the non-operation group in models I, II, and III (Table 3).

## Discussion

In this retrospective cohort study, we compared survival outcomes between surgical and non-surgical treatment in these patients using the SEER database. Firstly, we found that surgery and post-operative chemotherapy significantly prolonged survival, but triple therapy did not appear to benefit patients. Secondly, a larger proportion of patients did not receive surgery, mainly because doctors did not recommend this treatment for a variety of reasons. In addition, older patients with stage I PC who have not undergone surgery may benefit from chemotherapy, radiation, or chemoradiotherapy, with chemoradiotherapy benefiting the most.

Surgical treatment is the primary treatment option for patients with stage I PC. However, many factors affect the decision-making preference of elderly patients, such as physical weakness (17), poor rehabilitation ability (18), more comorbidities (19), and low motivation (20). Therefore, the need for surgery in these patients is a complex issue. Renz et al. (21) collected and analyzed the data of 300 patients who underwent partial pancreaticoduodenectomy or pylorus-sparing pancreaticoduodenectomy between 2002 and 2012. They found that a higher rate of pre-operative comorbidities in patients older than 75 years of age was associated with more post-operative non-surgical complications in this group (*p* = 0.002). However, the mOS (19.2 vs. 18.4 months) was not significantly different between the two groups. Moreover, among older patients with early-stage pancreatic cancer, 5-year overall survival was significantly higher in those who underwent surgery than in those who did not (25.0 vs. 2.3%; *p* < 0.0001), the median survival time was longer (24.3 vs. 5.8 months) (22). Similarly, other studies have confirmed the benefits of surgery (10, 23, 24). In a word, clinicians should not directly deny the operation plan of elderly patients according to the age of patients (9, 25) but need to carry out a rigorous and comprehensive evaluation of individual patients to maximize the survival benefit for operable elderly patients with stage I PC. Furthermore, we also compared the effect of different surgical modalities on prognosis and showed no difference in survival between distal pancreatectomy, partial pancreatectomy/pancreaticoduodenectomy, and total pancreatectomy.

Subsequently, we assessed the effectiveness of post-operative adjuvant therapy. Currently, adjuvant chemotherapy for resected PDAC is the standard of care after phase III clinical trials found a survival benefit associated with chemotherapy after surgical resection (3, 5, 26–30). However, the lack of randomized clinical trials in older adults, their suitability for treatment given limited life expectancy, and age-related changes in chemotherapy pharmacodynamics leading to increased toxic complications have led to uncertainty in treatment (31). King et al. (32) found that survival with chemotherapy and surgery was similar to that of the younger cohort (mOS: 20.3 months). Nagrial et al. (33) studied a cohort of patients who underwent surgical resection for pancreatic ductal adenocarcinoma and found that 30% of older patients received adjuvant therapy and that no chemotherapy was associated with worse survival (mOS: 13.1 vs. 21.8 months in treated patients). This trend of benefit exists even in elderly patients, but it is important to note that our study showed that adding radiotherapy to post-operative chemotherapy did not improve the prognosis of elderly patients with stage I pancreatic cancer, so the advantage of combined radiotherapy should be carefully evaluated in patients undergoing surgery.

Surprisingly, 81.4% of stage I patients in the study did not undergo surgery, mainly because surgery was not recommended, and past studies have had similar conclusion (34). Therefore, it is necessary to explore the effective non-surgical treatment for unoperated stage I pancreatic cancer. Zhu et al. (35) collected 100 patients with stage I PC who had not received surgical treatment from 2012 to 2016 and all received stereotactic radiotherapy, including 48 patients with induction chemotherapy and 52 patients with adjuvant chemotherapy. The results showed that patients in the adjuvant chemotherapy group had a longer survival (21 vs. 15 months, *p* = 0.001). They suggested that stereotactic radiotherapy in combination with adjuvant chemotherapy may be an alternative option for patients with resectable but medically inoperable pancreatic cancer. Another study (36) also showed that chemotherapy combined with radiotherapy can improve overall survival in patients with unresected PC. Most importantly, 55.1% of the people in the study were 65 years or older at the time of diagnosis, a whopping 3,389. Our study also found that the combination of chemoradiotherapy had the greatest benefit in inoperable resectable PC. As for patients who cannot tolerate combination therapy, stereotactic radiotherapy may be a good treatment option, even in the presence of serious complications (37). In addition, our study further found that the chemotherapy group and the radiotherapy group had the same prognosis, and the patients who chose palliative care had a worse prognosis. Hence, a more aggressive treatment approach should be carefully selected after evaluation by the clinician for those patients who have not had surgery.

This study has some limitations. Firstly, as a retrospective study, selection bias and confounding factors are inevitably disturbed. Although we used PSM and Cox regressions to try to compensate for these deficiencies, there are still some unidentified confounders and some known confounders that cannot be controlled. Secondly, the SEER database lacks detailed clinical information, such as physiological status score, resectable status, margin status, comorbidities, and post-operative recovery, which undoubtedly weakens the reliability of the conclusion of this study. It also did not provide patient preferences or patient characteristics (e.g., frailty) that might influence treatment decisions. These missing variables are critical for prognosis and need to be discussed in future studies.

## Conclusion

In conclusion, surgical resection and post-operative chemotherapy are recommended for elderly patients with early PC in the absence of surgical contraindications, but post-operative chemoradiotherapy does not bring survival benefits in elderly patients compared with post-operative chemotherapy. At the same time, radiotherapy, chemotherapy, or the combination of radiotherapy and chemotherapy are significantly related to the prognosis of elderly patients with unoperated PC, but chemoradiotherapy has the most obvious benefit. More large-scale clinical studies are needed to confirm our conclusion.

## Data availability statement

Publicly available datasets were analyzed in this study. This data can be found here: The Surveillance Epidemiology and End Results Program at https://seer.cancer.gov/.

## Ethics statement

Ethical review and approval was not required for the study on human participants in accordance with the local legislation and institutional requirements. Written informed consent for participation was not required for this study in accordance with the national legislation and the institutional requirements.

## Author contributions

DN designed the study and analyzed it. QL and BS wrote the main manuscript. DN and FX revised the manuscript. All authors finally revised and reviewed the manuscript.

## Footnotes

1
https://www.R-project.org/

## Supplementary material

Supplementary Table 1 Clinical characteristics of surgical and non-surgicalgroups in elderly patients with stage I pancreatic cancer after PSM.

Supplementary Table 2 Univariate and multivariate Cox analyses for elderlypatients with stage I pancreatic cancer after PSM.
