# Supplementary material for: Survival outcomes of surgical and non-surgical treatment in elderly patients with stage I pancreatic cancer: A population-based analysis
Source: Front Med (Lausanne). 2022 Sep 29;9:958257. doi: 10.3389/fmed.2022.958257 (PMC9556697; doi:10.3389/fmed.2022.958257)
Supplement: Supplementary Table 1 — Clinical characteristics of surgical and non-surgicalgroups in elderly patients with stage I pancreatic cancer after PSM. [file Table_1.DOCX]

| Variables | level | Overall (N=654) | No surgery (N=327) | Surgery (N=327) | P-value |
| --- | --- | --- | --- | --- | --- |
| Age (median [IQR]) |  | 74.0 [69.0, 79.0] | 73.0 [69.0, 79.0] | 74.0 [69.0, 79.0] | 0.813 |
| Age (%) | 65-74 | 350 (53.5) | 177 (54.1) | 173 (52.9) | 0.729 |
|  | 75-84 | 261 (39.9) | 131 (40.1) | 130 (39.8) |  |
|  | 85+ | 43 (6.6) | 19 (5.8) | 24 (7.3) |  |
| Sex (%) | Female | 347 (53.1) | 177 (54.1) | 170 (52.0) | 0.638 |
|  | Male | 307 (46.9) | 150 (45.9) | 157 (48.0) |  |
| Race (%) | Black | 61 (9.3) | 32 (9.8) | 29 (8.9) | 0.873 |
|  | Other | 73 (11.2) | 35 (10.7) | 38 (11.6) |  |
|  | White | 520 (79.5) | 260 (79.5) | 260 (79.5) |  |
| Year of diagnosis (%) | 2006-2011 | 306 (46.8) | 158 (48.3) | 148 (45.3) | 0.481 |
|  | 2012-2017 | 348 (53.2) | 169 (51.7) | 179 (54.7) |  |
| Grade (%) | I/II | 360 (55.0) | 177 (54.1) | 183 (56.0) | 0.855 |
|  | III/IV | 173 (26.5) | 87 (26.6) | 86 (26.3) |  |
|  | Unknown | 121 (18.5) | 63 (19.3) | 58 (17.7) |  |
| T (%) | T1 | 165 (25.2) | 81 (24.8) | 84 (25.7) | 0.857 |
|  | T2 | 489 (74.8) | 246 (75.2) | 243 (74.3) |  |
| Chemotherapy (%) | No/Unknown | 334 (51.1) | 173 (52.9) | 161 (49.2) | 0.390 |
|  | Yes | 320 (48.9) | 154 (47.1) | 166 (50.8) |  |
| Radiation (%) | None/Unknown | 551 (84.3) | 282 (86.2) | 269 (82.3) | 0.198 |
|  | Yes | 103 (15.7) | 45 (13.8) | 58 (17.7) |  |
