# Supplementary material for: Survival outcomes of surgical and non-surgical treatment in elderly patients with stage I pancreatic cancer: A population-based analysis
Source: Front Med (Lausanne). 2022 Sep 29;9:958257. doi: 10.3389/fmed.2022.958257 (PMC9556697; doi:10.3389/fmed.2022.958257)
Supplement: Supplementary Table 2 — Univariate and multivariate Cox analyses for elderlypatients with stage I pancreatic cancer after PSM. [file Table_2.DOCX]

| Variables | OS | | | | CSS | | | |
| --- | --- | --- | --- | --- | --- | --- | --- | --- |
|  | Univariate | | Multivariate | | Univariate | | Multivariate | |
|  | HR (95%CI) | P-value | HR (95%CI) | P-value | HR (95%CI) | P-value | HR (95%CI) | P-value |
| Age at diagnosis |  |  |  |  |  |  |  |  |
| 65-74 | Reference |  |  |  | Reference |  |  |  |
| 75-84 | 1.11 (0.94-1.32) | 0.222 | / |  | 1.08 (0.9-1.29) | 0.433 | / | / |
| 85+ | 1.14 (0.83-1.59) | 0.417 | / | / | 1.08 (0.75-1.54) | 0.683 | / | / |
| Sex |  |  |  |  |  |  |  |  |
| Female | Reference |  |  |  | Reference |  |  |  |
| Male | 0.97 (0.82-1.14) | 0.674 | / | / | 0.93 (0.78-1.11) | 0.445 | / | / |
| Race |  |  |  |  |  |  |  |  |
| Black | Reference |  |  |  | Reference |  |  |  |
| Other | 0.95 (0.66-1.36) | 0.768 | / | / | 0.99 (0.67-1.47) | 0.967 | / | / |
| White | 0.9 (0.68-1.20) | 0.484 | / | / | 0.97 (0.71-1.32) | 0.862 | / | / |
| Year of diagnosis |  |  |  |  |  |  |  |  |
| 2006-2011 | Reference |  | Reference |  | Reference |  | Reference |  |
| 2012-2017 | 0.71 (0.60-0.83) | <0.001 | 0.77 (0.65-0.91) | 0.002 | 0.68 (0.57-0.81) | <0.001 | 0.74 (0.62-0.88) | <0.001 |
| Primary site |  |  |  |  |  |  |  |  |
| Body/Tail | Reference |  | Reference |  | Reference |  | Reference |  |
| Head | 1.27 (1.04-1.55) | 0.018 | 1.36 (1.11-1.67) | 0.003 | 1.31 (1.06-1.63) | 0.013 | 1.4 (1.13-1.75) | 0.002 |
| Other | 1.22 (0.92-1.61) | 0.167 | 1.43 (1.08-1.90) | 0.014 | 1.26 (0.93-1.7) | 0.129 | 1.49 (1.1-2.02) | 0.010 |
| Grade |  |  |  |  |  |  |  |  |
| I/II | Reference |  |  |  | Reference |  | Reference |  |
| III/IV | 1.20 (0.99-1.46) | 0.059 | / | / | 1.27 (1.03-1.55) | 0.022 | 1.38 (1.12-1.70) | 0.002 |
| Unknown | 1.04 (0.83-1.29) | 0.755 | / | / | 1.01 (0.79-1.28) | 0.963 | 1.04 (0.81-1.35) | 0.748 |
| T stage |  |  |  |  |  |  |  |  |
| T1 | Reference |  | Reference |  | Reference |  | Reference |  |
| T2 | 1.29 (1.07-1.56) | 0.008 | 1.52 (1.25-1.86) | <0.001 | 1.35 (1.1-1.66) | 0.004 | 1.54 (1.23-1.93) | <0.001 |
| Surgery |  |  |  |  |  |  |  |  |
| None | Reference |  | Reference |  | Reference |  | Reference |  |
| Yes | 0.36 (0.30-0.42) | <0.001 | 0.35 (0.30-0.42) | <0.001 | 0.32 (0.27-0.38) | 0 | 0.31 (0.26-0.37) | <0.001 |
| Chemotherapy |  |  |  |  |  |  |  |  |
| None | Reference |  | Reference |  | Reference |  | Reference |  |
| Yes | 0.75 (0.63-0.88) | <0.001 | 0.68 (0.57-0.81) | <0.001 | 0.81 (0.68-0.96) | 0.016 | 0.71 (0.59-0.86) | <0.001 |
| Radiation |  |  |  |  |  |  |  |  |
| None | Reference |  |  |  | Reference |  |  |  |
| Yes | 0.93 (0.75-1.16) | 0.545 | / | / | 0.97 (0.77-1.22) | 0.775 | / | / |
